# The influence of workplace stressors on the risk of cardiovascular diseases among healthcare providers: a systematic review

**DOI:** 10.3389/fpsyt.2025.1461698

**Published:** 2025-08-21

**Authors:** Raghad Alhajaji, Manal Z. Alfahmi, Saleh Ahmed Alshaikhi, Abdulmajeed Mohammed Fairaq, Salman Fudlaldeen Jan, Sultan Aljuaid, Mashael AlFaifi, Mashael S. Alaboud, Imad Mohammed Khojah, Hadeel Alkofide, Khalid Al Sulaiman

**Affiliations:** ^1^ Makkah Branch of Ministry of Health, Ministry of Health, Makkah, Saudi Arabia; ^2^ Sleep Medicine Fellowship, King Saud University medical City, Riyadh, Saudi Arabia; ^3^ Executive Administration of Research and Innovation, King Abdullah Medical City, Makkah, Saudi Arabia; ^4^ Family Medicine Department, Jeddah Second Cluster, Jeddah, Saudi Arabia; ^5^ Public Health Authority Office, Makkah, Saudi Arabia; ^6^ Associate Executive Administration of Primary Health Care and Community Health, King Saud Medical City, Riyadh, Saudi Arabia; ^7^ Saudi Critical Care Pharmacy Research (SCAPE) Platform, Riyadh, Saudi Arabia; ^8^ Saudi Society for Multidisciplinary Research, Development, and Education, Riyadh, Saudi Arabia; ^9^ Pharmaceutical Care Department, King Abdulaziz Medical City, Jeddah, Saudi Arabia; ^10^ Department of Emergency Medicine, Faculty of Medicine, King Abdulaziz University, Jeddah, Saudi Arabia; ^11^ Department of Emergency Medicine, King Abdulaziz University Hospital, Jeddah, Saudi Arabia; ^12^ Department of Clinical Pharmacy, College of Pharmacy, King Saud University, Riyadh, Saudi Arabia; ^13^ Drug Regulation Research Unit, College of Pharmacy, King Saud University, Riyadh, Saudi Arabia; ^14^ Pharmaceutical Care Department, King Abdulaziz Medical City, Riyadh, Saudi Arabia; ^15^ College of Pharmacy, King Saud bin Abdulaziz University for Health Sciences, Riyadh, Saudi Arabia; ^16^ Clinical Trial Department, King Abdullah International Medical Research Center, Riyadh, Saudi Arabia

**Keywords:** job strain, cardiovascular diseases (CVDs), occupational stress, healthcare workers, coronary heart diseases, hypertension, shift work, long working hours

## Abstract

**Background:**

Cardiovascular diseases (CVDs) are a leading cause of death worldwide. Healthcare workers are at increased risk due to workplace stressors such as long hours, shift work, and high job demands, which may worsen both modifiable and non-modifiable CVD risk factors. This systematic review examines the impact of these workplace stressors on the risk for CVD among healthcare providers.

**Methods:**

We conducted a systematic review of observational studies from inception to January 2024, following PRISMA guidelines. We searched databases including PubMed, Embase, Scopus, Web of Science, and PsycINFO using keywords related to workplace stressors and CVDs among healthcare professionals. The quality of the studies was assessed using the Newcastle-Ottawa Scale (NOS).

**Results:**

Our review included 31 observational studies (15 cohort studies, 13 cross-sectional studies, and three case-control studies) with a total of 323,978 participants from 17 countries. The key stressors identified were long working hours, night shifts, and high job strain. Most studies reported significant associations between these stressors and increased risks of hypertension, ischemic heart disease, and cardiometabolic disorders. The quality of the studies ranged from fair to good, indicating a low risk of bias.

**Conclusion:**

Growing evidence suggests a strong correlation between workplace stressors and an increased risk of cardiovascular disease among healthcare workers. This leads to negative consequences that affect their performance and may extend to the quality of their patients’ care. Addressing these stressors through targeted interventions is crucial for protecting their health and improving patient care outcomes.

## 1 Introduction

The field of occupational health epidemiology began to investigate psychosocial work exposures in the 1990s (1). Since then, the body of literature has expanded significantly, making a synthesis of the existing research both timely and necessary (2). Healthcare professionals (HCPs) represent a high-risk occupational group due to the nature of their duties, which involve exposure to hazardous environments, rotating shifts, and work-related stress (3).

Work-related stress occurs when HCPs face job demands and pressures that surpass their abilities, leading to challenges in their performance (4). The stress becomes even more pronounced for HCPs when they sense a lack of support from their supervisors and colleagues, coupled with minimal control over their work processes (5). Moreover, various workplace situations can contribute to this stress, including the nature of the job, excessive workload, long working hours, limited opportunities for advancement, unclear or unfair performance reviews, poor communication, weak leadership, strained interpersonal relationships, and an insufficient work-life balance (6, 7).

The complexity between work-related stress and various health outcomes pose significant challenges for synthesizing research (8). Specifically, a substantial portion of the literature on psychosocial work exposures has focused on their associations with mental disorders and cardiovascular diseases (9–12). Cardiovascular diseases are the foremost cause of death worldwide. In 2019, they accounted for an estimated 17.9 million deaths—32% of all global deaths—with heart attacks and strokes responsible for 85% of these fatalities (13). Among HCPs, CVDs pose a significant health burden globally, accounting for approximately 50% of all deaths and 25% of work-related disabilities (14). A survey involving 2,500 physicians found significantly higher rates of cardiovascular disease risk factors, including stress, compared to a comparable sample from the general population (15).

Stress may promote cardiovascular disease by contributing to the development of metabolic syndrome—a cluster of at least three risk factors, including central obesity, hypertension, hyperglycemia, elevated triglycerides, and low HDL cholesterol (16). Metabolic abnormalities, in turn, accelerate atherosclerosis, characterized by the thickening of the arterial wall and the formation of plaques. Carotid intima–media thickness (IMT) serves as a noninvasive marker of early atherosclerotic change and can be measured using external ultrasound in extensive cohort studies (16). In one cross-sectional analysis of young adults, high job strain was linked to greater carotid IMT in men. However, this association was not observed in women, suggesting that occupational stress may differentially influence subclinical atherosclerosis by sex (17). Several lines of evidence support a causal relationship between workplace stressors and cardiovascular disease. First, the temporal sequence is precise, as exposure to work-related stress precedes the development of disease. Second, consistency across multiple studies reinforces this link, while biological plausibility is established by well-understood mechanisms through which stress can harm the cardiovascular system. The association also shows specificity, being most evident in particular heart and vascular conditions. Importantly, reversibility has been demonstrated: interventions that reduce job stress are associated with lower cardiovascular risk. Finally, the strength of the association—often with relative risks exceeding 2.0 for stressed versus unstressed workers—underscores the robustness of this effect (15, 16).

Several systematic reviews and meta-analyses revealed a significant association between work stress and the risk of cardiovascular disease (12, 18, 19). However, there is a scarcity of literature that has studied this association among HCPs. Therefore, this systematic review aimed to address the association between work-related stress and the risk of cardiovascular disease among HCPs.

## 2 Methods

### 2.1 Study design

This systematic review aims to investigate the impact of workplace stressors on the risk of cardiovascular diseases among healthcare providers. The Preferred Reporting Items for Systematic Reviews and Meta-Analyses (PRISMA) 2020 guidelines were adhered to throughout the study selection and data synthesis processes (11).

### 2.2 Search strategy

A comprehensive search strategy was developed using relevant keywords and Medical Subject Headings (MeSH) terms. Databases including PubMed, Embase, Scopus, Web of Science, and PsycINFO were systematically searched from inception to January 2024. Additionally, we hand searched the citation lists of the included studies and similar reviews for potentially eligible studies. The search strategy encompassed terms related to workplace stressors and cardiovascular diseases (among healthcare professionals consists of the following: (“Job Strain” OR “Job stress” OR “Work Strain” OR “Workplace Stress” OR “Occupational Stress” OR “Job Demand-Control” OR “Karasek”) AND (“Healthcare professionals” OR “Healthcare Workers” OR “Healthcare practitioners” OR “Physician” OR “Surgeons” OR “Nurses” OR “Medical Staff” OR “Pharmacists” OR “Medical assistants” OR “Healthcare administrators “OR “Health information technologists” OR “Dietitians” OR “Nutritionists” OR “Health educators” OR “Physical therapists” OR “Psychologists” OR “Paramedics” OR “Respiratory therapists” OR “Dentists”) AND (“Cardiovascular disease” OR “Cardiovascular event” OR “Coronary heart disease” OR “Acute Coronary Syndrome” OR “Myocardial Infarction” OR “Myocardial ischemia” OR “Heart Attack” OR “Angina” OR “Stroke” OR “Arrhythmias” OR “Ventricular Tachycardia” OR “Ventricular Fibrillation” OR “Acute Heart Failure” OR “Acute Aortic Dissection”). The search was limited to articles published in English to ensure relevance and comprehensiveness.

### 2.3 Study selection

The systematic review included observational studies with healthcare professionals as the population and workplace stressors such as job strain, long working hours, shift work, lack of social support, and other relevant factors as the exposure. The outcome evaluated was the risk of cardiovascular diseases, including angina, acute myocardial infarction, transient ischemic attack, and stroke. Studies focusing solely on burnout or job insecurity, including participants with baseline cardiovascular diseases, or not in the English language, were excluded from the review. Two independent reviewers screened the titles and abstracts of identified articles for eligibility based on predefined inclusion and exclusion criteria; a third reviewer was consulted to resolve any conflicts. Furthermore, full-text articles from potentially relevant studies were obtained and subsequently evaluated for eligibility by two independent reviewers. Any disagreement among the reviewers were resolved through discussion or by seeking input from a third independent author.

### 2.4 Data extraction and synthesis

Two authors independently extracted data using a standardized form. Extracted variables included: first author, year of publication, country, study design, sample size, population characteristics, type of workplace stressor, cardiovascular outcomes assessed, and key findings. Any disagreements were resolved either by reaching a consensus or by consulting a third reviewer. A narrative synthesis approach was employed to summarize the findings of included studies, considering the heterogeneity of study designs and outcomes.

### 2.5 Quality assessment

The quality of included studies was independently assessed by two reviewers using the Newcastle-Ottawa Scale (NOS) for cohort, cross-sectional, and case-control studies. Each study was rated based on selection, comparability, and outcome domains, with results tabulated in **Supplementary Table 2**. Disagreements were resolved either through consensus among the parties involved or by using a third-party adjudicator.

### 2.6 Ethics consideration

Ethical approval was not required for this study since it relied on existing literature.

### 2.7 Reporting

All methods employed in this systematic review, along with the inclusion criteria and the steps taken for data handling, have been thoroughly documented in accordance with PRISMA standards. This includes a detailed description of how studies were selected, the criteria used to evaluate their quality, and the strategies implemented for data extraction and analysis.

### 2.8 Definition of variables

Workplace Stressors: This includes any of the following exposures:

- Job strain: High psychological demands combined with low control, typically measured using the Job Content Questionnaire (JCQ) or Demand-Control-Support models.
- Long working hours: Defined as working 48 hours or more per week, or 55 hours or more based on WHO criteria.
- Shift work: Refers to non-standard work hours, including rotating shifts, night shifts, and continuous 24-hour shifts.
- Organizational stress: Includes stressors such as lack of managerial support, inadequate staffing or resources, poor communication, or workplace instability.
- Cardiovascular Disease (CVD) outcomes includes hypertension, ischemic heart disease, stroke or transient ischemic attack (TIA), and cardiometabolic syndrome.
- Hypertension (HTN): Identified through clinical diagnosis or based on blood pressure readings from ambulatory blood pressure monitoring (ABPM) or self-report.
- Ischemic Heart Disease (IHD): Includes angina, acute coronary syndrome, and myocardial infarction as confirmed by diagnostic criteria or medical records.
- Stroke or Transient Ischemic Attack (TIA): Reported based on imaging confirmation or physician diagnosis.
- Cardiometabolic risk factors: Includes obesity (defined as BMI ≥30), dyslipidemia, impaired glucose metabolism, and other indicators of metabolic syndrome.

## 3 Results

### 3.1 Search and screening

**Figure 1:**
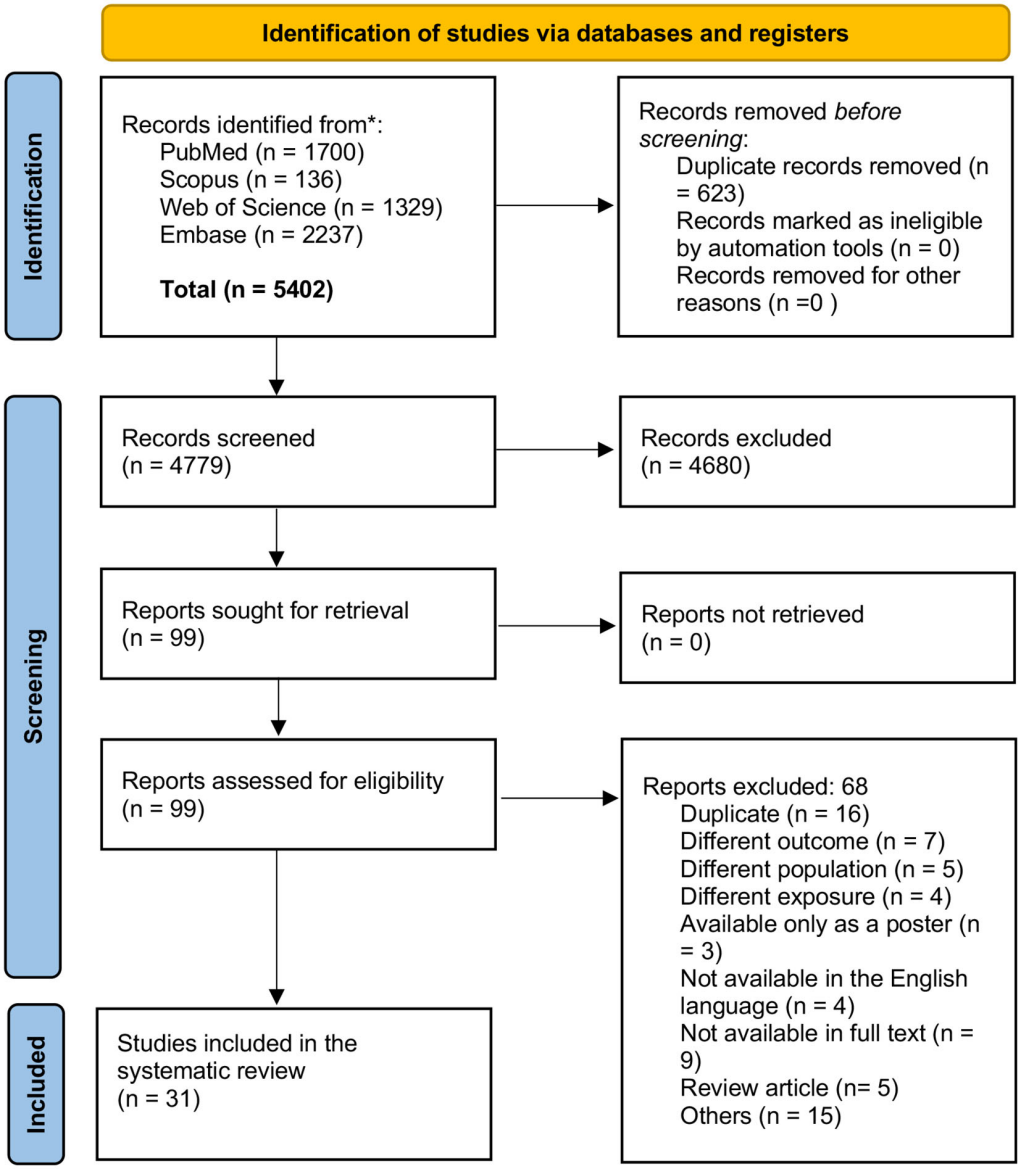
PRISMA flow diagram of the study selection process.

Our search and final screening process identified 31 observational studies, comprising 15 cohort studies, 13 cross-sectional studies, and three case-control studies, as illustrated in the PRISMA flow diagram (**Figure 1**). These studies encompassed a total of 323,978 healthcare workers across 17 countries, including the United States, Denmark, Japan, Canada, Serbia, Egypt, the Netherlands, Iran, Italy, Sweden, Taiwan, France, Turkey, India, Brazil, Norway, and Switzerland. The United States contributed the most significant number of studies (n = 6), followed by Brazil (n = 4) and Taiwan (n = 3).

This global distribution underscores both the widespread prevalence of workplace stressors in healthcare settings and the growing research interest in their cardiovascular consequences (**Table 1**).

**Table 1:** Baseline data of included studies.

Study ID	Country	Study design	Sample size	Follow-up	Age: range or mean (SD)	Sex: N (%)	Occupation	Baseline CVD risks	Cardiovascular domain
Allesøe, 2017 (18)	Denmark	Cohort	12,093	20.6 years	45–64	All females	Nurses	103 (0.9%) are Diabetic, 605 (5.0%) have a BMI over 30, and 4472 (37%) are current smokers	Ischemic heart disease
Adams, 1988 (12)	USA	Cohort	12	24 Hour	28-40	Females: 4 (33)	Emergency physicians	NR	Blood Pressure Variability and Heart Rate
Arnetz, 1998 (21)	Sweden	cross- sectional	66	NA	The mean age was 46.8 (7.9) years for the surgeons and 43.9 (7.0) for the general practitioners.	All males	General surgeons and GPs	Among the surgeons, four smoked less than 10 cigarettes per day, three smoked more than 10, and two smoked a pipe daily. Among the general practitioners, five smoked fewer than 10 cigarettes per day, and three smoked more than 10 cigarettes per day.	Not specified
Brown, 2003 (33)	USA	Cohort	59	24 Hour	Filipino-Americans: 33.7 (6.1) and Euro-Americans: 37.9 (6.6)	All females	Nurses and nurse’s aides	26.1% of Euro-Americans are Smokers, and 56.5% drink alcohol regularly	Blood Pressure Variability
Cash, 2021 (34)	USA	cross-sectional	379	NA	36 (10.1)	Females: 130 (36)	Emergency medical services employees	22% had hypertension 26 26 26 2 26%% had Hyperlipidemia, and 7% were Diabetic	Not specified
Chen, 2015 (20)	Taiwan	Cohort	28,062	NA	Median age: 46.81 (10.75)	Men: (85.72%)	Physicians	NR	Acute myocardial infarction
Chou, 2015 (41)	Taiwan	cross-sectional	1329	NA	21-64	Females: 1101 (82.8)	Physicians, Physician assistants, Nurses, and medical technicians	NA	Not specified
Chou, 2016 (28)	Taiwan	cross-sectional	576	NA	40.1 (8.4)	Females: 85%	Physicians, Nurses, Medical Technicians, Administrators	NR	Arteriosclerosis
Coelho, 2023 (36)	Brazil	cross-sectional	172	NA	42.7 (9.6)	Females: 149 (86.6)	Nurses	20.3% had hypertension	Blood Pressure Variability
Dutheil, 2017 (13)	France	Cohort	17	24 Hour	39.1 (6.9)	Females: 12 (63)	Emergency physicians	29% are smokers	Tachycardia
Ezber, 2023 (29)	Turkey	cross-sectional	160	NA	20–59	Females: 125 (78.1)	Doctors, nurses, and health care officers	40.% in particular had a Family history of CVD, 30% were smokers, and 15.6% drank alcohol regularly.	Not specified
Fatima, 2021 (14)	India	Cohort	50	24 Hour	36.7 (9.9)	All females	Nurses	NR	Blood Pressure Variability
Ferreira, 2021 (46)	Brazil	cross-sectional	324	NA	40.1 (8.7)	Females: 264 (81.5)	Nurses and nurses’ assistants	43.8% of participants were obese, 29.3% consumed alcohol, and 20.4% had hypertension	Not specified
Fialho, 2006 (15)	Brazil	Cohort	61	24 Hour	25.4 (1.43)	Males: 30 (53.6)	Medical residents	62.5% had a family history of hypertension, 44.6% had lipid disorders, and 35.7% had diabetes.	Blood Pressure Variability
Goffeng, 2018 (47)	Norway	Cohort	24	36 h	41.9(12.5)	Females: 21 (87.5)	Nurses and nurse assistants	NR	Heart Rate
Jensen, 2020 (24)	Denmark	Cohort	14,788	2 months	47 (10.64)	Females: 11,359 (77)	Doctors, Nurses, Dentists, social service, and administrative healthcare workers	NR	Ischemic heart disease and stroke
von Känel, 2023 (25)	Switzerland	Case-control	60	NA	Burnout group: 46.77 (10.56) and control: 52.93 (7.48)	All males	Physicians	Regular Alcohol consumption Mean (SD): 3.72 (3.22) in the burnout group and 2.93 (2.30) in the control group	Coronary microvascular function
Kubo, 2011 (16)	Japan	Cohort	36	24 Hour	32 (9)	All females	Nurses	NR	Coronary microcirculation
Lajoie, 2015 (31)	Canada	cross-sectional	271	NA	Night workers: 38.4 (11.6). Daytime workers: 45.0 (9.5)	All females	Nurses, Occupational therapists, physiotherapists, and radiation therapists	5.8% of Night workers were smokers, and 12.7% of daytime workers were smokers. 19.8% of night workers drink alcohol more than once a week, and the ratio was 36% among daytime workers	Cardio-metabolic status
Landsbergis, 2013 (27)	USA	cross-sectional	164	NA	23–76	Females: 72.6%	Nurses, Nurse assistants, physicians’ assistants, and other specialists	NR	Masked Hypertension
Nedić, 2010 (22)	Serbia	Case-control	109	NA	With Hypertension: 35–60 and Without Hypertension: 35–60	All females	Physicians	Both groups smoked 0–3 cigarettes per day. Three participants in each group consume alcohol	Blood Pressure Variability
Ramin, 2014 (32)	USA	Cross-sectional	54 724	NA	20–46 and more	All females	Nurses and nurse assistants	5% of day shift workers are smokers, and 7% of night shift workers are smokers. The mean (SD) g/day of alcohol consumption was 6.3 (10.3) among day workers and 6.3 (10.2) among night shift workers. Thirty-three participants have a history of hypertension among day workers and 36 among night shift workers.	Chronic diseases
Refaat, 2014 (17)	Egypt	Cohort	92	10 years	Male: 24.8 (2.9) and Females: 24.5 (3.4)	Females: 38(41.3)	Physicians	3.33% were smokers, and 2.22% had hypertension	Cardiometabolic status
Riese, 2000 (30)	Netherlands	Cross-sectional	165	NA	22-55	All females	Nurses	27.8% of the population were smokers	Cardiometabolic status
Riese, 2004 (38)	Netherlands	Cohort	159	12.2 months	35.9 (8.5)	All females	Nurses	Non-Healthy participants)	Ambulatory blood pressure, heart rate, and heart rate variability
Saberinia, 2020 (39)	Iran	Cross-sectional	250	NA	NR	NR	Nurses	23.6% of participants were smokers	Cardiometabolic status
Temporelli, 2013 (37)	Italy	Cross-sectional	1,770	NA	53	Females: 465 (26.5)	Physicians	49% of the participants had at least hypertension, hypercholesterolemia, active smoking status, diabetes, or previous CVD events.	Cardiometabolic status
Ulguim, 2019 (35)	Brazil	Cross-sectional	45	NA	30–39	Females 26 (57.8)	Healthcare workers	11.1% are current smokers	Cardiometabolic status
Vetter, 2016 (19)	USA	Cohort	189 158	24 years	30 - 55	All females	Nurses	Women in the NHS2 had slightly lower alcohol consumption and reported fewer pack-years of smoking	Coronary Heart Disease
Vitale, 2022 (26)	Italy	Case-control	60	NA	Cases: 41.1 (7.7) and the control: 40.3 (8.1)	Females: 24(40)	Physicians, nurses, and healthcare assistants	18% had a family history of CVDs	Cardiovascular Activity
Ramadan, 2024 (23)	Egypt	Cross-sectional	428	NA	28.22 (2.54)	Females: 224(52.3)	Physicians	15.2% have a history of migraine, and 6.1% have angina or heart attacks	Cardiovascular diseases and stroke

AMI, Acute Myocardial Infarction; BMI, Body Mass Index; BP, Blood Pressure; CVD, Cardiovascular Disease; EMS, Emergency Medical Services; GPs, General Practitioners; h, Hour(s); HR, Heart Rate; NA, Not Applicable; NR, Not Reported; SD, Standard Deviation; USA, United States of America; N (%), Number (Percentage); g/day, Grams per Day; BPV, Blood Pressure Variability; EMS, Emergency Medical Services; HRV, Heart Rate Variability; NHS2, Nurses’ Health Study II; N (%), Number (Percentage); SD, Standard Deviation; USA, United States of America.

### 3.2 Characteristics of included studies

The included studies varied significantly in design, population demographics, and methodological rigor. Participants primarily consisted of healthcare professionals such as physicians and nurses, nurse assistants, medical technicians, physiotherapists, dentists, social workers, and administrative staff. The most represented medical specialties were emergency physicians, general practitioners, surgeons, and cardiologists.

The age distribution among the study populations varied considerably, reflecting a broad spectrum of healthcare experience levels. Some studies focused on younger cohorts in their twenties, such as those by Adams et al. (12), Fialho et al. (15), Refaat et al. (17), and Ramadan et al. (23). Others included older individuals in their sixties, as seen in Allesøe et al. (18), Landsbergis et al. (27), and Chou et al. (28). The age ranges were extensive; for example, Ezber et al. (29) included participants aged 20–59 years, while Landsbergis et al. (27) had an age range of 23–76 years. Riese et al. (30) studied participants aged 22–55 years, and Vetter et al. (19) included individuals aged 30–55 years (**Table 1**).

Both genders (male and female participants) were represented across the included studies. Ten studies—such as those by Kubo et al. (16), Lajoie et al. (31), Nedić et al. (22), Ramin et al. (32), and Brown et al. (33)—investigated work strain exclusively among female participants. In contrast, only two studies, by Arnetz et al. (21) and von Känel et al. (25), focused solely on male participants. The remaining studies included both genders, providing a more comprehensive understanding of how work stressors affect male and female healthcare professionals differently (**Table 1**).

The studies investigated various workplace stressors, including job strain (characterized by high demands and low control), long working hours exceeding 48 hours per week, shift work (including night shifts and 24-hour shifts), heavy workload, organizational stress arising from workplace instability, rapid changes, or unhealthy work environments, and burnout—defined as chronic workplace stress resulting in physical and emotional exhaustion (25). The stressors were assessed using standardized questionnaires, including the Job Content Questionnaire (JCQ), Occupational Stress Index (OSI), and Perceived Stress Scale (PSS), as well as through interviews and workplace assessments.

The cardiovascular outcomes measured included hypertension, Ischemic heart diseases (IHDs), stroke, and cardiometabolic risk factors such as elevated lipid profiles, obesity, impaired glucose metabolism, and abnormal body mass index. Additionally, outcomes encompassed heart rate variability and coronary microcirculation. Assessment methods involved clinical measurements, physiological monitoring (e.g., ambulatory blood pressure monitoring, electrocardiography, echocardiography), and self-reported medical histories.

### 3.3 Follow-up durations

Regarding follow-up durations among the included studies, there were diverse temporal perspectives based on the outcomes and measurement tools. The follow-up periods vary from a span of 24 hours between the pre- and post-intervals, as seen in (12–16), to long-term follow-ups, such as the 10-year follow-up in (17), and even studies that extend over two decades, as illustrated in (18) and (19). This variability underscores the comprehensive exploration of outcomes across various time horizons, thereby enriching the depth and breadth of the synthesized evidence (**Table 1**).

### 3.4 Workplace stressors

Physicians are investigated to various forms of occupational stress, and the definition of work stressors differed across the included studies. Day-to-day work stress was the most commonly assessed type of job strain. Studies by Chen et al. (20), Arnetz et al. (21), and Allese et al. (18) reported additional stressors, including heavy workload, work influence, and occupational physical activity. The impact of long working hours on physicians was evaluated by Nedić et al. (22), while Fialho et al. (15) investigated stress associated with 24-hour shifts. Ramadan et al. (23) and Refaat et al. (17) examined the stressful lifestyle of medical residents over a decade, spanning from medical student level to practicing physicians. Stress related to organizational factors was assessed by Jensen et al. (24), and work-related burnout among physicians was explored by von Känel et al. (25). Additionally, Vitale et al. (26) specifically investigated work stress during afternoon shifts (**Table 1**).

### 3.5 Association between workplace stressors and cardiovascular outcomes

#### 3.5.1 Hypertension

Ten studies investigated the association between workplace stressors and HTN among healthcare workers, with most reporting a positive correlation. Adams et al. (12) conducted a cross-sectional study of emergency physicians and found that night shifts were associated with increased diastolic blood pressure (12). Similarly, Fialho et al. (15) reported that 24-hour shifts increased the prevalence of abnormal blood pressure readings among emergency medical residents (15). Nedić et al. (22) observed higher rates of hypertension among female physicians associated with long working hours (22). Arnetz et al. (21) identified a strong link between job stress and cardiovascular risk factors, including HTN, in a cross-sectional study (21). Brown et al. (33) found a significant association between job strain, catecholamine release, and elevated blood pressure among female nurses (33). Coelho et al. (36) reported that 20.3% of nurses had HTN, with work stress contributing to elevated blood pressure levels (36). Cash et al. (34) found that 22% of emergency medical services workers had HTN, with perceived stress being a significant predictor (34). Ulguim et al. (35) observed that work stress was associated with high blood pressure among nurses (35). Lajoie et al. (31) found an association between work stress and HTN in female nurses. However, Riese et al. (38), in a cohort study over a three-year follow-up, reported no significant association between high work strain and HTN among young female nurses (38). These findings suggest that specific stressors like night shifts and long working hours may contribute to elevated blood pressure, especially among female healthcare workers, highlighting the need for tailored interventions based on job role and gender (12, 15, 22, 33) (**Table 2**).

**Table 2:** Summary of included studies.

Study ID	Population and settings	Work risks	Objectives	Methods	Outcome measures	Main finding
Allesøe, 2017 (18)	Female nurses from the Danish Nurses Association	Influence at work and strenuous occupational physical activity	To investigate the association between occupational physical activity and IHD	12,093 nurses aged 45–64 followed for 20.6 years, linking to incident IHD in the Danish registry	Self-report	Work influence can offset the strain’s impact on heart disease risk, according to research findings.
Adams, 1998 (12)	EPs at an urban academic medical center	Occupational stress during the Night shift	To determine the relation between BP and HR and the night shift among EPs	Assessed BP and HRV in EPs during night shifts using monitors, comparing ED and non-ED periods	Oscillometric ambulatory BP device and Holter	Night shift raises blood pressure, suggesting stress-related changes and a heightened sympathetic tone. Urges further investigation for EP health.
Arnetz, 1988 (21)	General surgeons and GPs from a central registry managed by the organization for pharmaceutical information.	Working stress and long working hours	To investigate CVDs and psychosocial risk factors among surgeons and GPs	Structured questionnaire compared the cardiovascular and psychological status between GP physicians and general surgeons	Constructed Questionnaire	Strong links were found between job factors and conventional heart risk factors in the study.
Brown, 2003 (33)	Female nurses and nurse’s assistants who are Filipino American or Euro-American in ethnicity	Working frequency and strain	To examine the relationships between catecholamines and ambulatory BP during work stress	The study compared urinary catecholamine excretion and ambulatory blood pressure in Filipino-Americans and Euro-Americans.	Job Content Questionnaire, anthropometric, blood pressure, and catecholamine measurements	Work stress connects to heart risk; catecholamine excretion ties to BP variability. Cultural bias in job strain noted.
Cash, 2021 (34)	EMS employees from 4 county-based EMS agencies	Shift length and chronic stress	To investigate the associations between sleep duration and perceived and chronic stress during working hours	Surveyed CVD-free EMS personnel from 4 US agencies, assessing sleep quality, stress, chronic burden, and CVH components	The Pittsburgh Sleep Quality Index (PSQI), Perceived Stress Scale (PSS), Nic Burden Scale, and the CVH Measurements	EMS personnel: Adequate sleep is crucial for maintaining ideal cardiovascular health. Longitudinal studies are needed to explore sleep, stress, and CVD.
Chen, 2015 (20)	Physicians from Taiwan’s NHI Program and the general population	Heavy workload and work stress	To compare the risk of AMI between physicians and controls	Physicians’ data is sourced from the Medical Personnel Registry. AMI was identified via a computer algorithm using the ICD-9 code 410	Record based	Taiwanese physicians have higher HTN and hyperlipidemia rates but lower AMI risk. Medical centers offer better access to care.
Chou, 2015 (41)	Health-care workers from the health+promotion survey conducted at Sin-Lau Hospital (SLH-HPS).	Job strain	To investigate the risks of CVDs	Hospital-based survey in Taiwan with 1329 medical professionals (mean age 38). Assessed cardiovascular health using seven indicators and job strain.	The Seven Cardiovascular Health Indicators and the Chinese version of the Job Content Questionnaire,	Job strain is correlated with physical inactivity, particularly among nurses and physician assistants. Workplace health promotion should prioritize exercise motivation.
Chou, 2016 (28)	Volunteer healthcare workers from a regional hospital in Taiwan	Long working hours and demands	To investigate the relationships between arteriosclerosis and various work-stress conditions	Surveyed 576 medical employees (mean age 43, 85% female) in a regional hospital in Taiwan. Evaluated arteriosclerosis, work conditions, and mental health	Brachial-ankle pulse wave velocity (baPWV) and the Taiwanese Depression Questionnaire (TDQ)	Extended work hours and insufficient sleep raise the risk of arteriosclerosis. Employers and governments should regulate reasonable work hours to prevent heart disease.
Coelho, 2023 (36)	Nursing professionals from a large hospital in the state of Minas Gerais, Brazil	Night shifts	To assess risk factors associated with elevated BP	A quantitative study of 172 nursing professionals in a Minas Gerais hospital, Brazil. Data included anthropometric, BP, sociodemographic, clinical, and lifestyle factors. Analysis via bivariate and logistic regression with 5 5 5.5%5.5% significance level.	Digital sphygmomanometer, anthropometric data measurements, and electronic digital scale	Nursing workers: excess weight and night shifts linked to higher blood pressure levels.
Dutheil, 2017 (13)	EPs from the University Hospital of Clermont-Ferrand (CHU), France	Stress in Night Shifts	To investigate tachycardia and cardiac strain in different shifts	The trial monitored EPs’ HR with Holter-ECG in the shift-randomized study. Also assessed stress and emergencies	Holter-ECG. Visual analog scale	EPs experience high cardiac strain, especially in 24-hour shifts. Limiting such shifts is advisable. Heart rate monitoring is beneficial.
Ezber, 2023 (29)	Healthcare workers from a tertiary training and research hospital in Turkey	Day and night shifts	To highlight individual and occupational CVD risk factors and to warn healthcare professionals.	A study was conducted from March to September 2022, involving 160 participants, to assess sociodemographics, physical activity, work stress, and CVD risk.	Face-to-face interviews, international physical activity questionnaire, work stress scale, and SCORE	The study highlights the need to consider both work-related and individual risk factors for cardiovascular disease.
Fatima, 2021 (14)	Nurses working on critical-care units during night shifts in Era’s Lucknow Medical College and Hospitals.	Day and night shifts	To investigate the relationship between circadian rhythm, BP, and shift work	50 nurses (25 NSWN, 25 DSWN) had 24-hour ABPM and inflammatory markers measured, assessing CVD risk and circadian rhythm	Digital BP monitor	24-hour ambulatory blood pressure monitoring in NSWN revealed reduced circadian variation and abnormal rhythms compared to DSWN. Night shifts disrupt sleep, increasing cardiovascular risk.
Ferreira, 2021 (46)	Nursing at a public hospital in Minas Gerais	Day and night shifts	To assess CVD risks among nursing workers	Cross-sectional study with 324 nursing workers. Assessed sociodemographic, occupational, and health data, Framingham Risk Score, and psychosocial stress.	ABEP and DCSQ questionnaires	Ninety-six percent of workers showed a low 10-year cardiovascular disease risk, except for males over 40 with shorter work hours.
Fialho, 2006 (15)	Medical residents from Brazil in their first or second year of residency	24-h work shift	To investigate the effect of a 24-hour shift on BP	Sixty-one medical residents had ABPM during 24-hour ER shifts and common working days.	Mercury sphygmomanometer	ER residents on 24-hour shifts exhibit abnormal blood pressure, suggesting it is a cardiovascular disease risk.
Goffeng, 2018 (47)	Healthcare workers from the west coast of Norway	Extended work shifts	To investigate HR variability among 24-H healthcare workers	HRV is measured at work, leisure, and sleep. Parameters include time and frequency domains for analysis	The QPS Nordic Karolinska Sleepiness Scale and Bergen Insomnia Scale	Results show increased cardiovascular stress on the first working day compared to the fourth, with satisfactory recovery at night.
Jensen, 2020 (24)	Public healthcare employees from Denmark	Work organization and work stress	To investigate the relation between Work‐unit organizations and CVDs	Analyzed the risk of incident ischemic heart disease and stroke among 14,788 employees using survival models. Excluded pre-existing CVD	Record based	Organizational changes at work units may increase CVD risk compared to stable workplaces.
von Känel, 2023 (25)	Physicians from Switzerland	Burnout	To investigate the relationship between burnout, job stress, and coronary microvascular function.	Compared coronary microvascular function in 30 physicians with burnout and 30 controls. Assessed using myocardial perfusion PET	The Maslach Burnout Inventory and Job Stress Questionnaire, and myocardial tomography to assess the endothelium	Male physicians: burnout and high job stress affect coronary microvascular function differently. Longitudinal research is needed for clarity.
Kubo, 2011 (16)	Female Nurses from Japan	Day and night shifts	To investigate the effect of night-shift work on coronary microcirculation	Examined 36 female nurses post-nightshift and regular day with Doppler echocardiography. Calculated coronary flow reserve	Transthoracic Doppler echocardiographic examination	A study shows night shift work impairs coronary microcirculation in female nurses.
Lajoie, 2015 (31)	Healthcare workers from an acute-care teaching hospital in Southeastern Ontario, Canada	Day and night shifts	To identify the relationship between sleep disturbances and a cluster of CVD risk factors.	Compared female hospital employees on shift schedule with day-only workers. Assessed sleep quality and metabolic syndrome factors.	PSQI was used to assess sleep quality, and the MetS was defined according to the 2009 Joint Interim Studies consensus statement.	Rapid rotating shift women report poor sleep and high metabolic syndrome; sleep does not solely link to CVD.
Landsbergis, 2013 (27)	Hospital and home care employers in New York	Work-stressors	To assess the association between working conditions and masked hypertension.	45 male and 119 female hospital and home care employees wore ABPM during work hours—outcome: Masked hypertension defined by ABPM criteria. Associations with work stressors were tested by logistic regression.	Karasek’s Job Content Questionnaire and upper arm aneroid sphygmomanometer	Masked hypertension poses health risks. Further research on work-related factors is necessary for developing effective diagnosis, treatment, and prevention strategies.
Nedić, 2010 (22)	Physicians from the Novi Sad Clinical Center (NSCC).	long working hours and occupational stress	To examine the relationship between job stress and BP	Thirty-five female physicians with clinically diagnosed hypertension and 74 without completed the OSI questionnaire in Novi Sad.	OSI questionnaire and Mercury sphygmomanometer	Reducing work stress is crucial in preventing hypertension and other diseases, especially for female physicians
Ramin, 2014 (32)	Healthcare workers from Brigham and Women’s Hospital (Boston, Massachusetts, USA)	Night and day shift stress	To examine the relationship between night shift work and age, and risks of cancer and CVD, Age	socioeconomic status, and socioeconomic status-adjusted risk factors measured across night shift work categories. Logistic regression assessed associations among 54,724 participants, 72% ever shift workers, by age groups	NHS II questionnaire	Night shift work may increase the risk of chronic disease, with factors varying depending on the age of exposure.
Refaat, 2014 (17)	Fresh medical graduates who finished their training year at El-Minia University Hospitals	Medical profession strain	To evaluate the impact of medical work strain on CVD risk factors	Ninety-two medical graduates underwent baseline interviews, exams, and CVD risk assessments after ten years.	Electronic digital questionnaire	Moderate correlations between 2001 and 2011 values suggest that medical professional strain is associated with an increased risk of CVD. Stress coping and regular check-ups are recommended.
Riese, 2000 (30)	Nurses from three non-academic hospitals in Amsterdam	Job demands	To examine the relationship between job demands, decision latitude, and job-related social support on CVDs	Job strain measured by Job Content Questionnaire; CVD risk assessed by insulin, cholesterol, triglycerides, HDL-C, fibrinogen, tPA	The Dutch version of the JCQ	Studied job demands, decision latitude, and job-related social support on CVD risk in 165 female nurses.
Riese, 2004 (38)	Female nurses from the Vrije University, Netherlands	Job strain	To examine the relationship between job strain and CVDs	Studied 159 healthy female nurses (mean age 35.9) to minimize gender, SES, and work variance. Job strain, BP, HR, HRV assessed	The Dutch version of the JCQ. Ambulatory cardiac measures were obtained with the VU-AMS device	High job strain in young female nurses does not correlate with an unfavorable ambulatory cardiovascular profile. Gender differences in the impact of job strain on health are noted.
Saberini, 2020 (39)	Nurses from the Imam Khomeini Hospital of Tehran	Job stress	To find an association between occupational stress and risk factors for CVD	The study assessed occupational stress among 250 nurses at Emam Khomeini Hospital in Tehran, Iran, in 2018. Used the Osipow questionnaire	Osipow’s job stress questionnaire	High work-related stress affects blood glucose levels, but not other cardiovascular risk factors, among nurses.
Temporelli, 2013 (37)	Cardiologists from 3 national scientific societies in Italy	Work-stressors	To investigate CVD Profile and Lifestyle Habits	They used a web-based survey accessible via a dedicated site for data entry, which was then centralized in a database. The survey included four sections.	A Web-based electronic self-reported survey	Recent statements and guidelines suggest Italian cardiologists’ cardiovascular profiles are unlikely to be ideal.
Ulguim, 2019 (35)	Healthcare workers from the main hospital complex in Pardo River Valley, Brazil	Occupational stress	To identify risk factors for CVD and occupational stress	Cross-sectional study with 45 employees in Rio Grande do Sul, Brazil. Analyzed anthropometric, BP, biochemical markers, and occupational stress.	Anthropometric measurements, biochemical markers, and job stress were assessed by JSS.	The findings underscore the importance of health policies promoting lifestyle changes in and outside the workplace, thereby benefiting workers’ physical and mental well-being.
Vetter, 2016 (19)	Registered with the NHS and the NHS 2 studies	Night shifts	To determine the association between night shift work and CHD	Over 24 years, a prospective cohort study tracked 189,158 initially healthy women participating in the Nurses’ Health Study.	Biennial questionnaires were used to collect medical information from records.	Extended rotating night shifts raise CHD risk slightly in female nurses. Further research should examine specific work hours and individual traits.
Vitale, 2022 (26)	Healthcare workers from the emergency hospital in southern Italy	Afternoon shifts	To evaluate cardiovascular activity and emotional stress among Healthcare Workers during COVID-19	Studied HCWs’ cardiocirculatory activity and stress perception in COVID-19 wards. Included medical checks, ECGs, and questionnaires	Cardiac Holter device, ECG, SSPCS, and a ProQOL Questionnaire	COVID-19 ward work affects the blood pressure and heart function of healthcare workers. Attention is needed for cardiovascular risks.
Ramadan, 2024 (23)	Physicians from six university teaching hospitals in Egypt	Stressful lifestyle	To Investigate CVD and Stroke Risk Among Egyptian Resident Physicians	Surveyed six Egyptian university hospitals using a QRISK3 calculator to predict CVD and stroke over 10 years.	On-ground questionnaire developed (QRISK3 calculator)	Around 60.3% of resident physicians face heart attack or stroke risks within a decade. Urgent awareness and policy changes are needed.

ABPM, Ambulatory Blood Pressure Monitoring; AMI, Acute Myocardial Infarction; baPWV, Brachial-Ankle Pulse Wave Velocity; BP, Blood Pressure; BPV, Blood Pressure Variability; CHD, Coronary Heart Disease; CHU, Centre Hospitalier Universitaire (University Hospital Center); COVID-19, Coronavirus Disease 2019; CVD(s), Cardiovascular Disease(s); CVH, Cardiovascular Health; DBP, Diastolic Blood Pressure; DCSQ, Demand-Control-Support Questionnaire; DSWN, Day Shift Working Nurses; ECG, Electrocardiogram; ED, Emergency Department; EMS, Emergency Medical Services; EP(s), Emergency Physician(s); ER, Emergency Room; GP(s), General Practitioner(s); HCW(s), Healthcare Worker(s); HDL-C, High-Density Lipoprotein Cholesterol; HR, Heart Rate; HRV, Heart Rate Variability; HTN, Hypertension; IHD, Ischemic Heart Disease; ICD-9, International Classification of Diseases, 9th Revision; JCQ, Job Content Questionnaire; JSS, Job Stress Scale; MetS, Metabolic Syndrome; NHS, Nurses’ Health Study; NHS II, Nurses’ Health Study II; NHI, National Health Insurance; NSWN, Night Shift Working Nurses; OSI, Occupational Stress Index; PET, Positron Emission Tomography; ProQOL, Professional Quality of Life Scale; PSQI, Pittsburgh Sleep Quality Index; PSS, Perceived Stress Scale; QRISK3, A cardiovascular disease risk calculator; SCORE, Systematic Coronary Risk Evaluation; SD, Standard Deviation; SES, Socioeconomic Status; SLH-HPS, Sin-Lau Hospital Health Promotion Survey; SSPCS, Short Stress Perception Check Scale; TDQ, Taiwanese Depression Questionnaire; tPA, Tissue Plasminogen Activator; US, United States; VU-AMS, Vrije Universiteit Ambulatory Monitoring System.

#### 3.5.2 Ischemic heart disease

Eight studies explored the link between workplace stressors and IHD. Vetter et al. (19), in a 24-year cohort study of female nurses, found that night shift work was associated with a slight increase in coronary heart disease risk over time. Kubo et al. (16) reported that night shift work increased the risk of coronary heart disease during a 10-year follow-up among female nurses (16). Allesøe et al. (18) found that high job strain was significantly associated with IHD among female healthcare workers over two decades (18). Chen et al. (20) observed that high work stress was linked to an increased risk of IHD among healthcare workers over 8 years (20). Jensen et al. (24) discovered that unstable and continuously changing work environments were associated with a higher incidence of IHD compared to stable workplaces over an average follow-up of 5.5 years (24). Refaat et al. (17) found an increased risk of IHD associated with stressful work conditions among medical residents over 10 years (17). Von Känel et al. (25) identified that high job stress was associated with coronary microvascular dysfunction in male physicians (25). Ramadan et al. (23) reported that 60.3% of Egyptian physicians had experienced a heart attack or stroke, with a high prevalence among anesthesiologists and surgeons, attributing this to stressful lifestyles. Notably, several long-term cohort studies provided more substantial evidence of causality, with consistent findings across diverse populations and stressor types. Specifically, among female nurses exposed to prolonged night shifts or high job strain (16, 18, 19) (**Table 2**).

#### 3.5.3 Cardiovascular risk factors

Seven studies assessed the relationship between workplace stressors and cardiometabolic risk factors. Refaat et al. (17) observed that stressful lifestyles among medical residents were associated with increased obesity and dyslipidemia over a 10-year follow-up (17). Riese et al. (30) found that high job strain was associated with elevated lipid profiles and a BMI reading indicating overweight and obesity among healthcare workers over three years (30). Saberinia et al. (39) reported a significant correlation between work stress and impaired glucose metabolism among nurses in a cross-sectional study (39). Temporelli et al. (37) found that work stress was linked to increased obesity and components of metabolic syndrome among healthcare professionals (37). Ulguim et al. (35) observed that high work stress correlated with adverse cardiometabolic profiles, including elevated cholesterol levels (35). Chou et al. (28) reported an association between work-related stress and overweight or obesity among healthcare workers (28). Fatima et al. (14) found that job stress was linked to elevated blood glucose levels among healthcare staff. Although methodologies and outcomes varied, a consistent trend emerged linking psychosocial stress to adverse metabolic profiles, reinforcing the hypothesis that chronic occupational stress contributes to CVD development via cardiometabolic pathways (17, 30, 39). (**Table 2**).

#### 3.5.4 Other outcomes

Additional cardiovascular outcomes were examined in several studies. Dutheil et al. (13) found that 24-hour shifts were associated with increased heart rates and reduced heart rate variability among physicians, indicating heightened sympathetic activity (13). Kubo et al. (16) reported that night shift work adversely affected coronary microcirculation among nurses, as assessed by transthoracic Doppler echocardiography (16). Adams et al. (12) observed that night shifts were associated with increased heart rate and blood pressure variability among emergency physicians (**Table 2**).

### 3.6 Outcome differences according to the study design

#### 3.6.1 Results from cross-sectional studies

Thirteen cross-sectional studies evaluated various workplace stressors and their association with cardiovascular outcomes among healthcare professionals. The nature of their study design limits their ability to prove causality.

The primary workplace stressors examined were job strain, long working hours, and organizational stress. For instance, Brown et al. (33) and Nedić et al. (22) used standard tools like the Job Content Questionnaire and the Occupational Stress Index to measure job stress and found strong links between it and high blood pressure and a higher risk of CVD (**Table 2**). Vitale et al. (26) focused on work-related burnout, specifically investigating the impact of afternoon shift work on physician health. Notably, Ramadan et al. (23) found that 60.3% of Egyptian physicians had experienced a heart attack or stroke, with a higher prevalence among anesthesiologists, surgeons, and cardiologists, attributing this to stressful work environments.

#### 3.6.2 Results from cohort studies

The 15 cohort studies included in this review provide more robust evidence regarding the causal relationship between workplace stressors and the development of CVDs. Workplace stressors included long-term exposure to high job demands, extended working hours, night shifts, and organizational factors. Chen et al. (20) and Allesøe et al. (18), for instance, investigated the impact of prolonged work stress on cardiovascular health. Allesøe et al. (18) reported that female healthcare workers exposed to high job strain over a follow-up period exceeding two decades emphasize a significantly increased risk of IHD (**Table 2**). Vetter et al. (19) demonstrated that night shift work was associated with a gradual increase in CVD risk among female nurses throughout careers. Similarly, Refaat et al. (17) monitored medical residents over 10 years, with a high incidence of HTN and CVD linked to stressful work conditions.

### 3.7 Quality and risk of bias assessment

The quality assessment of the three case-control studies reveals that Vitale et al. (26) stands out with a Good quality rating, scoring 8 out of 9. This study met all key selection criteria, including an adequate case definition, representativeness of cases, and proper control selection and definition. It also controlled for confounders and adequately assessed outcomes with sufficient follow-up, indicating a well-designed study with minimal risk of bias. In contrast, von Känel et al. (25) earned a Fair rating with five points (stars), falling short in the selection of controls and in assessing outcomes rigorously. While the study controlled for confounders, incomplete reporting on control selection and outcome assessment may introduce potential biases, limiting the generalizability of its findings. Similarly, Nedić (22), also rated Fair, and received six points (stars). Although the study provided an adequate case definition and outcome assessment, it lacked representativeness in case selection and control selection, which poses a moderate risk of bias. Nonetheless, it adequately follows up with participants and offers valuable insights despite these limitations. (**Supplementary Table 3**). Regarding cross-sectional studies included in this review, Arnetz et al. (21) and Ramadan (23) both received an eight (Good) rating, excelling across key categories, such as representativeness of the sample, sample size, and exposure ascertainment. These studies also controlled for confounding factors and employed appropriate statistical tests, indicating a high level of methodological rigor. Studies like Cash (34), Chou (41), and Riese (30), scoring seven (Good), maintained solid designs but lacked in areas like sample representativeness or handling of non-respondents. Nonetheless, they ensured robust exposure assessments and conducted thorough statistical analyses. However, some studies, such as Coelho (36) and Lajoie (31), were rated Fair with scores of six due to weaknesses in managing non-respondents or comparability between groups. Both Ezber (29) and Temporelli (37) received Poor ratings with scores of four, suffered from significant issues, including inadequate handling of non-respondents, lack of control for confounders, and limited statistical validation. (**Supplementary Table 2**).

The cohort studies in this review demonstrate varying levels of methodological rigor, as evaluated by the Newcastle-Ottawa Scale (NOS). Chen (20) and Riese (38) both received 8 (Good) scores, indicating a strong design across selection, comparability, and outcome assessment. These studies controlled for confounders effectively, ensured that the outcome of interest was not present at the study’s start, and had adequate follow-up, making them highly reliable in assessing cohort-related outcomes. Allesøe (18), Fatima (14), and Jensen (24) scored seven (Good), showing good representativeness and robust follow-up procedures, though they had minor limitations in their exposure ascertainment or selection of the non-exposed cohort. On the other hand, several studies, such as Adams (12), Fialho (15), and Vetter (19), received Fair scores, ranging from 5 to 6, due to weaker selection processes or failure to adequately demonstrate that the outcome of interest was absent at the study’s initiation. Brown (33) and Dutheil (13), scoring five (Fair), also faced issues in their assessment of confounders and follow-up adequacy, which could compromise the reliability of their result.] (**Supplementary Table 1**).

## 4 Discussion

The systematic review identified 31 observational studies comprising 15 cohorts, 13 cross-sectional, and three case-control studies, illustrating diverse 323,978 healthcare workers with different professions across 17 countries, with follow-up durations varying from 24 hours to multi-year spans. Work stressors among healthcare workers were multifaceted, including job strain, long working hours, night shifts, 24-hour shifts, and organizational stress. Studies have evaluated work strain across different medical professions and age cohorts. Both genders were represented in the studies, although some investigations focused exclusively on either males or females. The methodologies used to assess work strain and cardiovascular diseases varied, showcasing a range of approaches from questionnaires to physiological measurements. Baseline risk factors for cardiovascular diseases, such as smoking and hypertension, were reported across multiple studies. The majority of studies found strong associations between job stress and hypertension, ischemic heart diseases, and cardiometabolic status among healthcare workers.

Cardiovascular diseases (CVDs) are the leading cause of death among the whole population in both developed and developing countries. According to the WHO, CVDs were responsible for approximately 17.9 million deaths during 2019 (40). Several factors can precipitate CVDs; modifiable risks include smoking, alcohol, obesity or dyslipidemia, stressful or sedentary lifestyle, while non-modifiable CVD risks include a family history of CVDs, age, and gender (23).

Healthcare workers, besides being liable to both types of CVD risks, are vulnerable to both stressful lifestyles and stressful working strains, making them more liable to develop CVDs. The included studies have found a significant number of physicians, nurses, and other healthcare employees suffering from CVDs or have a great risk of developing coronary heart disease (16, 19, 25).

Various theories have explained the association between job stressors in medical professions and CVDs. Long working hours, night shifts, work influence, and toxic work environments are significant work stressors that have been shown to induce CVDs (18, 20, 21). The high influence of work stressors leads to constant attention, lifestyle disturbances, and psychological stress (36). Chronic psychological workplace stress is positively correlated with ischemic heart disease (41). Also, the lack of sleep during night shifts is significantly correlated with developing CVDs as a result of circadian rhythms desynchronization and blood pressure variations (36).

Medical specialty among physicians is also a risk factor; emergency physicians (EPs) are frequently liable to physiological stress due to their on-calls and long working hours, which may lead to poor cardiac health and CVDs (34). Adams et al. (12) found that EPs had higher blood pressure during night shifts; furthermore, Dutheil et al. (13) found that EPs experienced higher heart rates, especially in 24-hour shifts; these findings may be explained by sleep deprivation and sympathetic activity. Also, Ramadan et al. (23) found that 78.3% of Anesthesiology specialists were correlated with CVD events, followed by surgery residents who exhibited 62.9%. This result is anticipated, as the extended work hours characteristic of select medical specialties are often associated with significant psychological stress for those practitioners, which can lead to a lack of awareness regarding their physical health and, consequently, an increased incidence of cardiovascular diseases among these populations.

The included studies found a strong association between job stress and hypertension, especially during night shifts (12, 21). Which may be linked to catecholamine release (33). Hypertension was common during 24-hour shifts and among female physicians (15, 22).

One important consideration is the significant heterogeneity among the studies included in this systematic review. The variability in study design, population characteristics, and the tools used to measure both workplace stressors and cardiovascular outcomes may have influenced the overall findings. For instance, the differences in defining and assessing job strain, long working hours, and shift work across studies introduce potential biases that could affect the comparability of the results. Similarly, the range of follow-up durations, from short-term assessments over 24 hours to multi-year longitudinal studies, may lead to inconsistent conclusions regarding the long-term cardiovascular effects of workplace stressors. This diversity across the studies makes it challenging to draw definitive conclusions across all studies, and caution is needed when generalizing the findings to broader populations of healthcare workers.

Ischemic heart diseases were also reported widely in the included studies; in a multi-centric cross-sectional study, Ramadan et al. (23) interestingly found that around 60.3% of Egyptian physicians suffer from a heart attack or stroke, and the included specialties were anesthesiologists, ICU, surgery, rheumatology, internal medicine, cardiology, oncology, pediatrics, obstetrics, and gynecology. Night shifts were associated with a higher risk of coronary heart diseases, especially among female nurses (16, 19) and among male physicians (25). Besides other CVD risks, the unstable work environment might be the key responsible factor, as reported by Jensen et al. (24).

While this review notes that some studies did not find a significant association between work strain and CVDs (42–44), other studies have found the contrast (16, 19, 24, 25). It is essential to examine the underlying reasons for these inconsistencies. Differences in study design, population characteristics, and stress measurement methods are likely contributing factors. For example, Riese et al. (30) included young nurses with age ranges from 25 to 50, meaning that the population may not have been exposed to enough chronic work strain. Another explanation proposed by Allesøe et al. (18) is that women in postmenopausal periods are more likely to be affected by work strain due to the lack of protective effects of estrogen. Hence, younger female physicians may be unlikely to be affected. However, this explanation is arguable, as a study by Laflamme et al. (45) found that older women are unlikely to be associated with a positive correlation between work strain and CVDs.

Additionally, the methods used to assess stress—ranging from self-reported questionnaires to physiological measures, such as heart rate variability—vary widely in their sensitivity and precision. Some studies may have used less reliable or validated tools, potentially underestimating the actual impact of work strain. Furthermore, cultural and healthcare system differences in the studied countries may influence job stress levels and the diagnosis and treatment of CVDs, potentially explaining the variability in the results. These factors highlight the need for more standardized methodologies and extended follow-up periods in future research to better understand the relationship between work strain and CVD among healthcare workers.

According to our findings and the literature, physicians and other healthcare workers are at a considerable risk of CVDs. It is essential to recognize that physicians’ health is closely tied to the quality of patient care. The risk of making medical errors is increasing in their practice due to attention failures (20).

Furthermore, physicians are reluctant to seek medical care, especially from their colleagues. They are afraid to feel weak or compromised, so they tend to continue working despite work strain and ignore potential symptoms, which can later impact their health condition and the quality of care they provide to patients.

Several studies included in our review had relatively small sample sizes, which notably limited the statistical power and generalizability of their findings. For instance, Adams et al. (12) conducted a cohort study in the USA involving only 12 emergency physicians (33% female; ages 28–40 years), assessing blood pressure variability and heart rate over 24 hours. Arnetz et al. (21) performed a cross-sectional study in Sweden with 66 male general surgeons and general practitioners (mean age approximately 44–47 years). Brown et al. (33) studied 59 female nurses and nurse’s aides (ages 33.7–37.9 years) in the USA, focusing on blood pressure variability. They noted that 26.1% of the Euro-American participants were smokers and 56.5% consumed alcohol regularly (33). Similarly, Dutheil et al. (13) investigated tachycardia among 17 emergency physicians in France (63% female; mean age 39.1 years), finding that 29% were smokers. Other studies with small samples include Fialho et al. (15) with 61 medical residents in Brazil (53.6% male; mean age 25.4 years), assessing blood pressure variability and reporting a high prevalence of family history of hypertension and lipid disorders (15); Fatima et al. (14) with 50 female nurses in India (mean age 36.7 years), examining blood pressure variability (14); Kubo et al. (16) studying coronary microcirculation in 36 female nurses in Japan (mean age 32 years); Ulguim et al. (35) assessing cardiometabolic status among 45 healthcare workers in Brazil (57.8% female; ages 30–39 years), with 11.1% being smokers (35); and Vitale et al. (26) examining cardiovascular activity in 60 healthcare workers in Italy (40% female; mean age around 41 years), noting that 18% had a family history of cardiovascular diseases (26).

The review also revealed several methodological gaps across studies. Definitions of exposure (e.g., “long working hours”) and outcomes (e.g., “cardiometabolic risk”) were not always consistent. Follow-up durations varied significantly, and not all studies adequately controlled for confounding factors such as age, baseline comorbidities, and lifestyle behaviors.

Moreover, the impact of gender and profession-based differences (e.g., physicians vs. nurses) have not been sufficiently investigated in numerous studies. We suggest that future research should establish standardized definitions, include the analysis of stress biomarkers, and utilize consistent criteria for cardiovascular outcomes. Additionally, there is an urgent need for interventional studies that evaluate the effectiveness of workplace adjustments, such as changes in schedules and the implementation of support systems, in lowering the risk of cardiovascular disease.

## 5 Conclusion

Emerging evidence demonstrates an association between occupational stress in healthcare professionals and elevated cardiovascular risk. Contributing factors include extended working hours, night and 24-hour shift schedules, high job strain, and adverse workplace environments. These factors have been linked to increased risk of hypertension, ischemic heart disease, and other cardiometabolic disorders. Beyond the direct impact on physical health, these stressors may also compromise clinical performance and patient safety. Targeted interventions and sustained research efforts are critical to mitigate these risks, promote the well-being of healthcare workers, and maintain the quality and resilience of healthcare systems.

## 6 Limitations and recommendations

Despite the importance of CHDs among healthcare workers, it was inapplicable to pool the studies and perform a meta-analysis due to the majority of the included studies have included mixed samples of healthcare workers from different professions (Physicians, nurses nurse assistants, etc.) and, the measurement tools and questionnaires were different among studies, and the lack of CHDs specifications among studies. Hence, we recommend a future organized study investigating profession-specific studies on physicians, nurses, and medical technicians, with medical specialty subgroup analyses (e.g., emergency medicine, surgery, anesthesiology). Furthermore, we should utilize standardized and validated questionnaires to gauge the impact of job stress on the cardiovascular system. Future research should identify the categories of diseases under study to accurately link workplace stressors to cardiovascular disorders such as hypertension, ischemic heart disease, and arrhythmias. Additionally, we require multicenter, cross-cultural, and longitudinal studies with extended follow-up periods. Longitudinal research can determine how job stressors affect cardiovascular health over time.

Furthermore, future studies should incorporate a comprehensive stressor assessment to ascertain the impact of specific work stressors, such as long hours, night shifts, shift rotations, workload, and organizational challenges, on cardiovascular health. This also involves examining the relationships between psychological stress, burnout, job satisfaction, and work-life balance, and their impact on physical stressors and cardiovascular health, as well as implementing a study comparing stress biomarkers. Additional research on cost-benefit analyses aiming to assess the effectiveness of the possible interventions to reduce work-related stress and prevent CVDs is recommended, targeting the underrepresented healthcare workers, including allied health professionals, support staff, and those in low- and middle-income countries.

## Data Availability

The original contributions presented in the study are included in the article/**Supplementary Material**. Further inquiries can be directed to the corresponding author.

## Author’s note

Protocol registration: The protocol for this review has not been recorded in PROSPERO or any other registries.

## Glossary

ABEP: Associação Brasileira de Empresas de Pesquisa
AMI: Acute Myocardial Infarction
BMI: Body Mass Index
BP: Blood Pressure
CHD: Coronary Heart Disease
CI: Confidence Interval
CVD: Cardiovascular Disease
CVDs: Cardiovascular Diseases
DCSQ: Demand-Control-Support Questionnaire
DM: Diabetes Mellitus
ECG: Electrocardiogram
EMS: Emergency Medical Services
EP: Emergency Physician(s)
GP: General Practitioner(s)
HCWs: Healthcare Workers
HR: Heart Rate
HTN: Hypertension
ICU: Intensive Care Unit
IHD: Ischemic Heart Disease
IHDs: Ischemic Heart Diseases
JCQ: Job Content Questionnaire
JSS: Job Strain Scale
MeSH: Medical Subject Headings
MetS: Metabolic Syndrome
NHS: National Health Service
NHS2: Nurses’Health Study II
NOS: Newcastle–Ottawa Scale
OSI: Occupational Stress Index
PICO: Population, Intervention, Comparator, Outcome
PRISMA: Preferred Reporting Items for Systematic Reviews and Meta-Analyses
PROSPERO: International Prospective Register of Systematic Reviews
ProQOL: Professional Quality of Life Scale
PSS: Perceived Stress Scale
QRISK3: Cardiovascular Disease Risk Assessment Tool
RR: Relative Risk
SCORE: Systematic Coronary Risk Evaluation
SD: Standard Deviation
SSPCS: Social Stigma towards Patients due to COVID Scale
USA: United States of America
WHO: World Health Organization
